# Evaluation of EMG pattern recognition for upper limb prosthesis control: a case study in comparison with direct myoelectric control

**DOI:** 10.1186/s12984-018-0361-3

**Published:** 2018-03-15

**Authors:** Linda Resnik, He (Helen) Huang, Anna Winslow, Dustin L. Crouch, Fan Zhang, Nancy Wolk

**Affiliations:** 1 Health Services, Policy and Practice, School of Public Health, Brown University, 121 South Main Street, Providence, RI 02908 USA; 2 Providence VA Medical Center, Providence, RI USA; 3 Joint Department of Biomedical Engineering, North Carolina State University, Campus Box 7115, Raleigh, NC 27695 USA; 4 Joint Department of Biomedical Engineering, University of North Carolina at Chapel Hill, 150A MacNider Hall, Chapel Hill, NC 27599 USA; 5 Department of Mechanical, Aerospace, and Biomedical Engineering, University of Tennessee, Knoxville, TN USA; 6 Rex Hospital, Raleigh, NC USA; 7 Closed-Loop Engineering for Advanced Engineering (CLEAR) Core, North Carolina State University, Campus Box 7115, Raleigh, NC 27695 USA

**Keywords:** Myoelectric control, Upper limb prosthetics, EMG pattern recognition, Direct control, Transradial amputees; case report

## Abstract

**Background:**

Although electromyogram (EMG) pattern recognition (PR) for multifunctional upper limb prosthesis control has been reported for decades, the clinical benefits have rarely been examined. The study purposes were to: 1) compare self-report and performance outcomes of a transradial amputee immediately after training and one week after training of direct myoelectric control and EMG pattern recognition (PR) for a two-degree-of-freedom (DOF) prosthesis, and 2) examine the change in outcomes one week after pattern recognition training and the rate of skill acquisition in two subjects with transradial amputations.

**Methods:**

In this cross-over study, participants were randomized to receive either PR control or direct control (DC) training of a 2 DOF myoelectric prosthesis first. Participants were 2 persons with traumatic transradial (TR) amputations who were 1 DOF myoelectric users. Outcomes, including measures of dexterity with and without cognitive load, activity performance, self-reported function, and prosthetic satisfaction were administered immediately and 1 week after training. Speed of skill acquisition was assessed hourly. One subject completed training under both PR control and DC conditions. Both subjects completed PR training and testing. Outcomes of test metrics were analyzed descriptively.

**Results:**

Comparison of the two control strategies in one subject who completed training in both conditions showed better scores in 2 (18%) dexterity measures, 1 (50%) dexterity measure with cognitive load, and 1 (50%) self-report functional measure using DC, as compared to PR. Scores of all other metrics were comparable. Both subjects showed decline in dexterity after training. Findings related to rate of skill acquisition varied considerably by subject.

**Conclusions:**

Outcomes of PR and DC for operating a 2-DOF prosthesis in a single subject cross-over study were similar for 74% of metrics, and favored DC in 26% of metrics. The two subjects who completed PR training showed decline in dexterity one week after training ended. Findings related to rate of skill acquisition varied considerably by subject. This study, despite its small sample size, highlights a need for additional research quantifying the functional and clinical benefits of PR control for upper limb prostheses.

**Electronic supplementary material:**

The online version of this article (10.1186/s12984-018-0361-3) contains supplementary material, which is available to authorized users.

## Background

In the United States, there are approximately 41,000 persons who have major upper limb amputations [1]. Activities that a person could routinely perform may no longer be possible or require additional effort and time following upper limb amputation [2]. Upper limb prostheses can assist amputees in activities of daily living (ADLs) such as feeding, dressing, and hygiene tasks. These performance capabilities are highly desired by persons with upper-limb amputation, regardless of their amputation level or current prosthesis type [3]. Unfortunately, due to the limitations of clinically available prosthetics technologies, a substantial proportion of persons with upper limb amputation (10–25%) do not use a prosthesis [4–7]. Of those patients that do use a prosthesis, only approximately 50% of subjects use an electric prosthesis [8]. Improving prosthetic technology in ways that yield appreciable benefits in tasks that amputees identify as important, such as ADLs, is essential to increase acceptance rates of electric prostheses and, ultimately, improve quality of life post-amputation.

The advances in mechatronics in recent years have resulted in mechanically complex prosthetic arm systems, which permit control across multiple degrees-of-freedom (DOFs) typical of intact arms [9–11]. For example, multifunctional hands possess great potential to restore the dexterity of the missing hand in upper limb amputees [9–11], and some of them have become commercially available, i.e. the i-Limb (Össur, Iceland) and bebionic (Otto Bock, Germany). The DEKA arm, an advanced prosthetic arm recently approved by the FDA, has a fully powered 3 degree-of-freedom (DOF) shoulder, elbow, 2-DOF wrist, and 6 hand gripping patterns [12].

Nevertheless, almost all commercial electric prostheses, including modern dexterous hands, still use a ‘direct myoelectric control’ approach, first offered commercially in the 1970s [13]. For the direct control (DC) approach, EMG signals recorded from a residual agonist-antagonist muscle pair are used. One muscle controls one direction of the motor in a prosthetic joint. The motor speed is proportional to the magnitude of the EMG signal [14, 15]. Control of multiple DOF devices using DC is challenging for several reasons. First, two residual muscles must be activated independently in order to drive one prosthetic joint, yet sometimes it is difficult to localize two independent EMG recording sites on amputees. Second, direct control becomes non-intuitive when the number of prosthetic joints that must be controlled increases. For example, when two or more DOFs are externally powered, the user must use a special muscle activation pattern (such as co-contraction) to switch the control joint before using EMG signals to proportionally drive the selected joint. This method can also be used for the dexterous hand to select hand grip patterns by toggling through several grips, a process which is time consuming and cumbersome. Finally, the DC approach requires training users to generate appropriate muscle activation patterns and manually setting the threshold of EMG magnitude for control [16].

The state-of-the-art myoelectric control for multifunction prostheses is based on EMG pattern recognition (PR) [17–19]. This approach has just become commercially available (COAPT, IL). The assumption underlying EMG PR-based control is that EMG signals recorded from multiple residual muscles or targeted reinnervated muscles [20, 21] produce different patterns when the amputee attempts different movements in the missing arm. Based on EMG activation patterns, pattern classifiers can identify the user’s intended hand and wrist motions (e.g. hand grip patterns, hand open, supination, pronation, etc.) with over 90% classification accuracy [22]. Compared to conventional ‘direct myoelectric control’, EMG PR-based control is expected to lead to more intuitive multifunctional prosthesis operation during everyday activities.

Despite the promise of EMG PR-based prosthesis control, its functional benefits during tasks typical of daily living, as well as its clinical viability are still unclear. In the engineering research community, classification accuracy/error rate in identifying user intent has been commonly reported to demonstrate the promise of EMG PR algorithms for prosthesis control [23–25]. The EMG PR was also evaluated while the users performed virtual tasks or selected physical tasks [22]. Many of these evaluations were conducted on able-bodied individuals. It is unclear how these laboratory metrics and the results derived from able-bodied individuals translates to comprehensive clinical evaluation tasks and daily activities for upper-limb amputees. Basic movements required by amputees include various grasping arrangements, environment interactions, and manipulation about various DOFs [3]. Yet, complex tasks like ADLs involve combinations of grasping and manipulation tasks [3], and there is limited research assessing PR-based control in this context.

In addition, many studies of PR control have focused on engineering approaches (such as study of data sources, features, and classifiers) in order to increase EMG PR accuracy and reliability in identifying user intent [26–29], while very limited research has been conducted to quantify the efficacy of EMG PR-based prosthesis control as compared to clinically available direct myoelectric control. Therefore, the answers to clinically relevant questions have not been addressed. Does EMG PR offer more functional benefit to amputee users (the target population)? Is EMG PR more acceptable to amputee users? Does the cost of EMG PR outweigh the function regained? In our opinion, answering these questions is critical to inform further engineering adjustments for EMG PR-based control. We are aware of only one recent study that compared two myoelectric control methods for a 3 degree-of-freedom transradial (TR) prosthesis by 3 transradial amputees [30]. That study showed that, after home trials, PR control yielded better control for tasks requiring wrist function. Although this study is timely and important, it is insufficient evidence to generalize conclusions.

Given the dearth of literature that directly compares EMG PR to clinically available direct EMG control, additional evidence is needed to compare the benefits of EMG PR to DC for clinical use. The primary purpose of this study was to compare EMG PR-based control and DC of a 2 DOF electric prosthesis on transradial amputees for functional performance of real-word tasks. A secondary purpose was to examine the change in outcomes one week after EMG PR training and the rate of skill acquisition in two subjects with transradial amputations. Evaluation protocols from early-stage case studies like Kuiken et al. [30] and our work, which quantified outcomes of dexterity with and without cognitive load, activity performance, self-reported function, and prosthetic satisfaction, could serve as a basis for future research studies comparing PR control and DC.

## Methods

### Study design

The experimental design involved a cross-over study with participants randomized to receive either PR control or direct control training first.

### Participants

This study was conducted with the approval of the Institutional Review Board (IRB) at the University of North Carolina at Chapel Hill, and informed consent was obtained for all subjects. We included individuals with transradial amputations caused by traumatic injuries, with evidence of two viable myoelectric sites as determined by prosthetist screening or successful prior use of dual site direct control (DC). Persons were excluded if their residual limb length prohibited socket fitting or if they had significant uncorrectable visual deficits, major communication or neurocognitive deficits, skin conditions preventing prosthetic wear, electrically controlled medical devices, severe circulatory problems or cognitive or mental health problems that would limit full participation in the study. Persons with prior experience in DC *with mode switching* were also excluded. By chance, all recruited subjects in this study were myoelectric prosthesis users prior to this study who were familiar with DC although they may have used DC to drive only a single DOF of their usual prosthesis. All subjects were naïve to pattern recognition (PR) prosthesis control. Although we considered limiting our study to participants who had no experience with either DC or PR control (which might provide more fair comparison), we decided not to impose this constraint given that (1) the amputees most likely to use PR control in the future are current DC users, (2) the constraint would further challenge our capability for recruiting amputees due to the small size and geographic dispersion of the population, and (3) the participants are naïve to using DC for multi-joint prosthesis operation; they still need to learn how to switch between control mode (joint) and how to operate other DOFs *non-intuitively* using hand open/close intent.

Although four subjects with transradial amputations were recruited for this study, the first two subjects were fit with the experimental prosthesis and began PR-based control training, but withdrew prior to completing training. Thus, these subjects were not tested and will not be discussed in this paper. Of the remaining two subjects, one subject (Subject TR4) completed the full protocol, which included training and testing with both EMG PR and DC conditions. The final subject (Subject TR3) completed PR control training and testing.

Subject TR3 was a 46-year-old male who had lost his limb 3 years prior to study participation due to a work-related accident. At the time of enrollment he used a myoelectric prosthesis consisting of a manual wrist rotator and an i-Limb terminal device. He controlled hand open/close with EMG and could select 3 grasping patterns with single, double, or triple quick muscle twitches. Subject TR3 participated in 7 h of PR control training from 5/5/16–6/7/16, after which he was tested twice, one week apart. The subject sustained an injury un-related to the research study and was unable to return to complete the cross-over portion of the study.

Subject TR4 was a 27-year-old female who had lost her limb due to a motor vehicle accident approximately one year prior to study participation. At the time of enrollment TR4 used a myoelectric prosthesis with an i-Limb terminal device. She had a manual wrist rotator and was able to switch between 3 grasp patterns with single, double, or triple quick muscle twitches. She participated in 11 h of DC training from 7/11/16–8/11/16 after which she completed testing with DC, and she repeated testing one week later. She then crossed over to train with PR and underwent 12 h of PR training from 9/15–11/7/2016 before completing two PR control test sessions with a one-week break between sessions.

### Experimental setup

A 2-DOF transradial prosthesis was used in this study. The prosthetic device included a commercial wrist rotator (MC Wrist Rotator, Motion Control, Inc., USA) and an active terminal device (ETD, Motion Control Inc. USA). These 2 DOFs (wrist pronate/supinate and terminal device open/close) were selected because most transradial amputees who use myoelectric devices only use one or two active joints, and these are the ones most commonly used. The prosthesis was mounted on the experimental socket, which was customized for each subject. Six active EMG electrodes (TRIAD Preamp, Motion Control, Inc., USA) were embedded within the experiment socket for surface EMG signal measurement. Two electrodes were used for DC and all six electrodes were used for PR control. The gain for each electrode was set by inspecting the display of EMG signals in real time on a computer screen. Both DC and EMG PR were implemented on our own computing processor (Texas Instruments OMAP3503 600 MHz processor based on the ARM Cortex-A8 architecture) [25]. EMG signals were sampled at 1000 Hz and digitally filtered between 20 and 450 Hz. Two of six electrodes were placed on the DC sites identified by a certified prosthetist. These two EMG electrodes were placed over the residual limb, where independent EMG activations were recorded while amputees attempted wrist/finger flexion and extension. The gain for each channel was then manually set by the prosthetist in order to use the entire dynamic range of the recorded EMG signal. Four additional EMG electrode locations were selected by palpating residual limb muscles and checking EMG recordings when the subjects were instructed to perform hand open/close and wrist pronation/supination. Note that since EMG crosstalk has little influence on the performance of EMG PR [31], targeting specific muscles for EMG electrode placement was not necessary.

### Prosthetic control configurations: pattern recognition and direct control

#### Direct control

EMG signals recorded from the 2 DC electrode sites were used as the input for DC. Signal magnitude was first estimated by calculating the mean absolute value (MAV) of 50 ms samples of EMG data. If the magnitude of one muscle was larger than a predefined threshold, a corresponding prosthetic motor was activated; the speed of the motor was proportional to the magnitude of the EMG signal. The proportional control gain was adjusted until both subjects were satisfied with the motor speed that matched their own prosthesis control. The thresholds were defined by the prosthetist so that any muscle crosstalk or low-level co-activation did not cause erroneous movements. If the magnitudes of both EMG signals were above threshold values (detected as muscle co-contraction), the prosthesis control mode (either wrist rotator or prosthetic hand) was switched. That is to say, forearm muscle co-contraction was used to switch between DOFs. This calibration procedure for DC was conducted once for each individual participant. Fine tuning of the DC control parameters was allowed at the beginning of each DC training/testing session based on subject feedback.

#### EMG pattern recognition

**Fig. 1:**
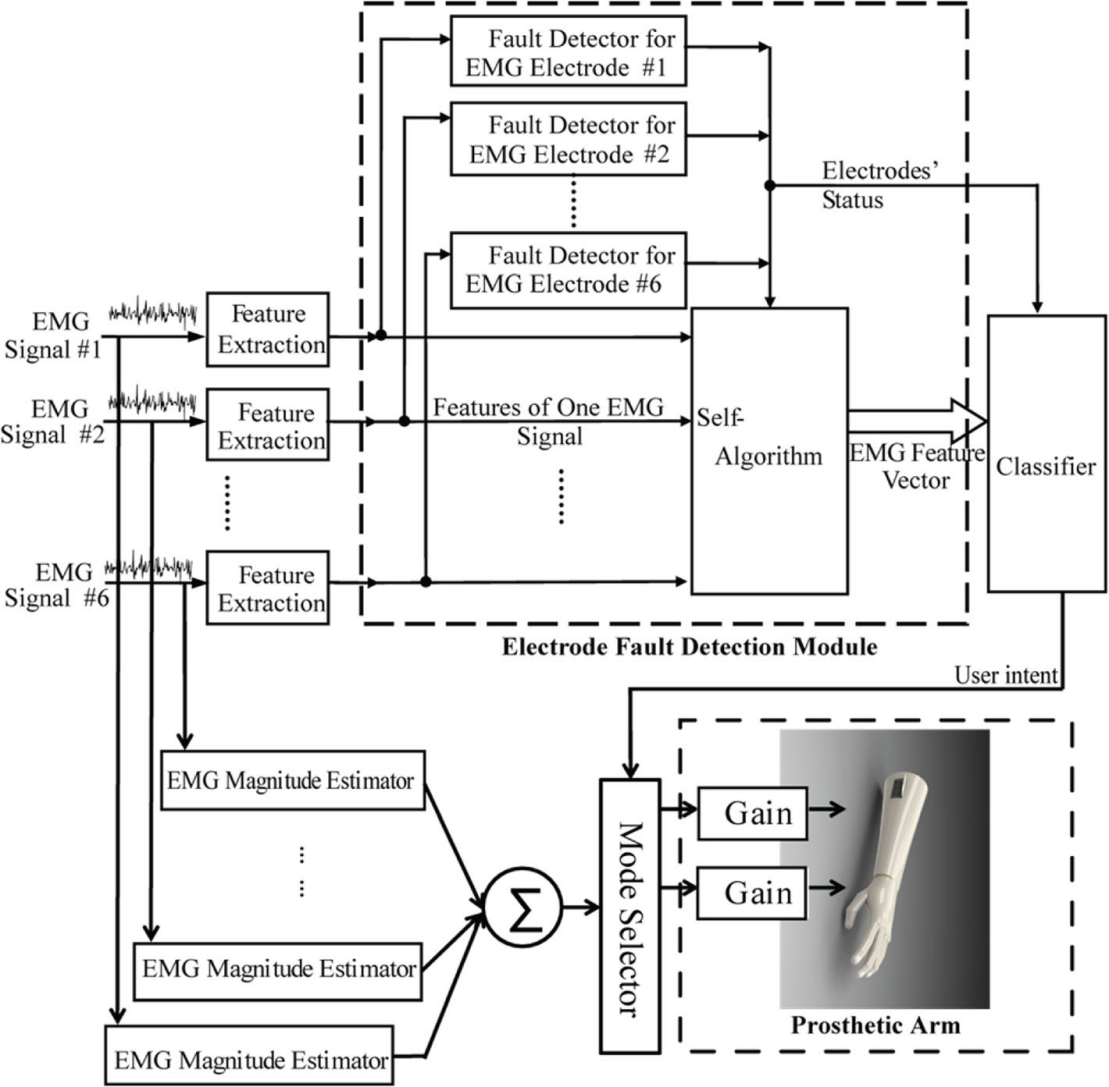
Architecture of EMG pattern recognition-based prosthesis control used in this study

The architecture of EMG PR control applied in this study is shown in Fig. 1. The input EMG signals were streamed into the system and analyzed within overlapping, sliding time-windows (the window length was 200 ms; the window increment was 50 ms). In each window, four commonly used time-domain (TD) features (MAV, number of zero crossings, waveform length, and number of slope sign changes), which represent the pattern of EMG signals, were extracted from each input channel. The detailed calculation of these features can be found in a previous publication [32]. All features were organized into a single vector. One limitation of EMG PR is the lack of robustness to disturbances at the sensor interface [25, 33–36]. To better handle potential signal disturbances and improve control robustness, we included a sensor fault detection module that detected faults in the input signals [25]. If an EMG recording provided abnormal readings, the features of the signal were removed from the feature vector for classification in order to ensure reliable system performance. Finally, the feature vector in each window was fed into a linear discriminant analysis (LDA)-based classifier [37]. The classifier determined the user’s intended movement. There were four active classes of movement (hand open, hand close, wrist pronation, and wrist supination) and one inactive class (no movement). The LDA classification decision was passed to a prosthesis motor selector, which activated the motor according to the intended movement. The motor speed was proportionally driven by the sum of magnitudes of all the EMG signals. The detailed engineering design, implementation, and evaluation of our applied PR control were reported in our previous study [25].

A calibration procedure^1^ was conducted in order to establish the parameters of the EMG classifier. This procedure was necessary every time right after the subject donned the prosthesis. In the calibration procedure, subjects sat in front of a computer monitor comfortably. A computer graphic interface (GUI) instructed the subjects to attempt to perform one of 5 studied motions in the missing wrist/hand and hold the motion for 3 s. The shoulder was around neutral position; the elbow was flexed approximately 90 degrees. Each motion was repeated twice at the beginning. Motions were separated by 3 s of rest. EMG signals (input) and attempted motion (output) were recorded. The known input-output data were then used to calibrate the classification boundary in the LDA-based classifier. Once this procedure was completed, we asked the subject to perform the 5 studied motions at different shoulder positions (including combinations of shoulder flexion at 45, 90, or 135 degree and shoulder abduction/adduction at 45, 0, or − 45 degree) when the elbow was fully extended or flexed to approximately 90 degrees. Different postures were tested because previous studies showed that EMG PR performance decreased with changes of upper limb posture [38]. If obvious EMG PR errors were observed at a certain upper limb posture, additional training data were collected at that posture for the EMG PR calibration. The system recalibration was allowed during the experiments. This was because EMG PR lacked robustness against EMG variations caused by electrode shift, electrode-skin impedance change, muscle fatigue, or other environmental or physiological factors [33, 35], which sometimes led to decreased performance over time.

### Experimental protocol

After the initial visit to confirm eligibility and collect baseline data, subjects were seen by the study prosthetist who fabricated the prosthetic socket and worked with the engineering team to set-up prosthetic controls for the initial condition. Upon the completion of socket fitting, the subject began training sessions with the study occupational therapist. The occupational therapist was an experienced therapist (> 20 years of clinical experience), who was new to EMG PR training. Prior to beginning the study she was trained by the engineers in the lab to understand how EMG PR works and experienced control of a virtual arm using EMG PR. She was also trained to administer the testing and training protocol by one of the authors (LR). She utilized a standardized protocol developed for the study by LR (See Additional file 1).

**Fig. 2:**
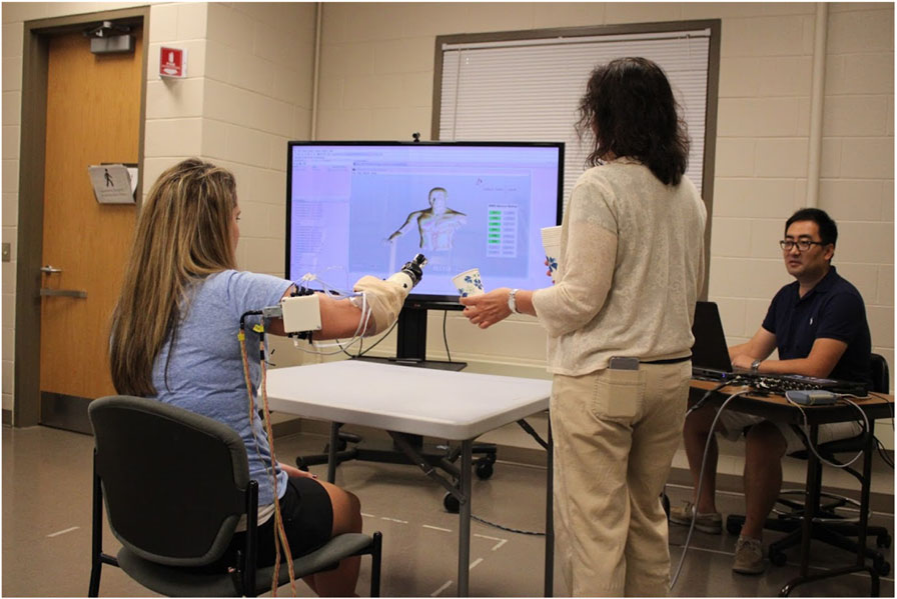
Subject training with system in virtual reality

The exact length and frequency of training sessions varied due to subject availability and travel schedule. Training sessions were 2 h or less in length. The intervals between two training sessions ranged between 2 and 7 days. Subjects were provided with regular rest periods to avoid fatigue. The protocol called for 5 min of rest every 30 min, or more if necessary to minimize fatigue. Training activities began with basic control training using virtual reality (VR) with the subject wearing the experimental prosthetic socket and prosthesis (with the prosthesis disabled). Virtual reality training continued until the subject was able to demonstrate moderately consistent command of controls for each of the four movements (hand open, hand closed, supination, pronation) using a virtual avatar (Fig. 2). Training then progressed to controls training wearing the prosthesis. Tasks included the grasp and release of objects with various shapes and sizes and basic dexterity activities. As subjects gained greater proficiency, they progressed to bilateral activities and more complex functional activities (see Additional file 1). Training activities were performed in close to the body, and also in varying positions including: extended in front of the body, extended with arms to the side, and with arms overhead. If, after initial calibration, the subject developed difficulty with the operation of any movement (due to fatigue or socket shifting) recalibration or re-donning the prosthetic socket was performed.

Once training with the actual prosthesis commenced, subjects were administered the Jebsen Taylor Hand Function (JTHF) page- turning test [39] at 1 h intervals to assess the ease of learning and speed of mastery of the new EMG PR control system as compared to EMG DC. The test took around 2 min to administer. Note that the subjects were not allowed to practice the JTHF prior to test administration. JTHF page turning test results (scored as the number of items completed per second) were analyzed to determine if the subjects’ progress had plateaued and training should be terminated. The decision to end training was made if the JTHF page turning results, in items/s, were stable over 3 consecutive testing results defined as performance less than the minimal detectable change at 90% confidence (MDC 90) for this measure, which is 0.11 items/s. Otherwise, the subject continued training sessions until he/she demonstrated consistent performance on the JTHF page- turning task or reached a maximum of 20 h of training. For both PR control and DC, subjects achieved consistent performance on the JTHF page-turning task in 12 h of training or less. At the conclusion of training, study tests and measures were administered. The subject returned to the study site approximately one week later to be retested in order to assess retention of skill. The goal for this follow-up examination was to quantify short-term retention of skill acquisition. Retention of motor learning is commonly done in studies of motor learning and has implications for prosthetic rehabilitation protocols [40, 41].

### Evaluation metrics

Evaluation metrics included performance-based and self-report measures (Table 1). Performance metrics included the Box and Block test [42], the modified Jebsen-Taylor Hand Function (JTHF) test [43], the Activities Measure for Upper Limb Amputees (AM-ULA) [44], the Clothes Pin Relocation Task [45], and the Clothes Pin Relocation Task with dual-task performance where the subject repeats the clothes pin task while simultaneously performing a cognitively demanding task: 1) counting backwards by 3 s from 100, and 2) naming as many fruits and vegetables as possible, and the differences in timed performance of these tests is calculated and considered as the impact of cognitive load.

**Table 1:** Description of study measures

Test	What it measures	Content	Scoring	Interpretation	MDC 90
Patient-Specific Functional Scale (PSFS)	Difficulty performing activities	5 self-selected activities difficult to do because of the amputation	Likert scale 0–10 0 = unable to perform 10 = can perform activity easily	Higher scores indicates less difficulty	6.49
Upper-Extremity Functional Scale (UEFS)- modified	Difficulty performing activities	Self-reported difficulty performing 22 everyday activities	Likert scale 1–5 1 = very easy 5 = cannot perform	Lower scores indicates less difficulty	12.07
Clothespin relocation task	Dexterity	Number of clothespins moved in 1 min	Count	Higher score indicates better dexterity	Unknown
Clothespin relocation with counting backwards	Dexterity with cognitive load	Number of clothespins moved in 1 min while counting backwards from 100 by 3 s	Count	Reduction of # clothespins shows the impact of cognitive burden on performance	Unknown
Clothespin relocation with semantic fluency	Dexterity with cognitive load	Number of clothespins moved in 1 min while naming as many fruits and vegetables as possible	Count	Reduction of # clothespins shows the impact of cognitive burden on performance	Unknown
Trinity Amputation and Prosthesis Experience Scales (TAPES)	Prosthetic satisfaction	10 items satisfaction with prosthesis	Likert scale 1–5 1 = very dissatisfied 5 = very satisfied	Higher scores indicate greater satisfaction	0.79
Jebsen-Taylor Hand Function Test (JTHF)	Dexterity		Count of completed items/sec	Higher scores indicate better performance	
Writing		Writing 24 letter sentence	“	“	0.18
Page Turning		Turning over index card	“	“	0.11
Small objects /sec		Picking up small objects	“	“	0.09
Simulated Feeding		Using a spoon to pick up and place small objects in can	“	“	0.10
Stacking checkers		Stacking checkers	“	“	0.11
Moving light cans		Moving light cans	“	“	0.15
Moving heavy cans		Moving heavy cans	“	“	0.13
Activities Measure for Upper-Limb Amputees (AM-ULA)	5 elements of performance: completion: speed, movement quality, skill and independence	18-everyday tasks	Likert scale 0–5 0 = unable 5 = excellent	Higher scores indicate better performance	3.7

Self-report measures included the Upper Extremity Functional Scale (UEFS) from the Orthotics and Prosthetics Users Survey (OPUS) [46, 47], the Patient Specific Functional Scale (PSFS) [48, 49], which has been used in the VA studies of the DEKA Arm, and the satisfaction scale from the Trinity Amputations and Prosthetics Experience Scale (TAPES) [50]. Tasks identified as difficult by the subjects for the PSFS assessment included tying shoes, holding a drink, picking up and moving objects (e.g. ladder), carrying items weighing less than 10 lb. at their side, putting on a bracelet, drying their hair, putting their hair in a ponytail, buttoning, and turning a rotating tray.

### Data analysis

Data from Subject TR4 was used to compare outcomes of the two types of control schemes. Data from both Subject TR3 and Subject TR4 were used to evaluate 1-week retention in dexterity and activity performance for EMG PR control. Data from Subjects TR3 and TR4 were used to examine the rate of skill acquisition for EMG PR control as measured by the JTHF page turning test. This test was administered at the end of each hour of training for each condition. That said, this test was not attempted in Subject TR4 for the PR condition until after the 8th hour because until that time the training session was focused on using virtual reality and re-creating reliable patterns. Given that this was a case series, differences in scores were analyzed descriptively, and inferences about magnitude of differences were made using known values of MDC 90 when known.

## Results

### Comparison of PR control and DC

Comparisons of outcome measures administered immediately after the completion of PR control training and DC training (Test 1) are shown in Table 2. The subject performed better on the Box and Blocks and the JTHF writing test with direct control as compared to PR. The differences between other JTHF tests, AM-ULA and UEFS were within the margin of error (as determined by the MDC 90 of the measures (Table 2)). The subject also performed better in the clothespin relocation task with DC as compared to PR under all conditions and appeared to be less impacted by cognitive load caused by naming fruits and vegetables (semantic fluency) with DC as compared to PR (MDC 90 for this test unknown). Further, the subject reported less difficulty in task performance (PSFS) when reporting on DC as compared to PR control (MDC 90 unknown). There were no differences in TAPES satisfaction ratings between control types.

**Table 2:** Outcomes for Pattern Recognition and Direct Control immediately after training and one week later: Subject TR4

Evaluation		PR control tesT 1	DC test 1	PR control test 2	DC test 2
Box and Blocks		7	17^a^	15	21
JTHF Tests (items/sec)	Writing	0.47	0.89^a^	0.84	0.91
	Page Turning	0.21	0.15	0.10	0.16
	Small Objects /sec	0.08	0.06	0.05	0.08
	Simulated Feeding	0.09	0.17	0.07	0.15
	Stacking Checkers	0.14	0.23	0.17	0.18
	Moving Light Cans	0.38	0.34	0.45	0.32
	Moving Heavy Cans	0.30	0.19	0.32	0.20
Clothespin Relocation (# pins moved)		6	11	3	7
Clothespin Relocation with counting backwards		6	10	5	5
Clothespin Relocation with semantic fluency		4	11	6	8
AM-ULA		15.0	13.5	13.3	15.6
PSFS Total		4.6	5.8	4.6	5.2
UEFS Score		43.00	47.00	46.00	45.00
TAPES Total		3.2	3.2	3.1	NT

*NT* not tested

^a^Differences between PR and DC exceed known MDC 90

Comparisons of outcome measures administered one week after concluding PR control and DC training (Test 2) show that there were no differences between PR control and DC in the Box and Blocks, any JTHF test or the UEFS. Differences in self-reported difficulty scores (PSFS) persisted one week after testing, with the subject reporting less difficulty in task performance with DC as compared to PR.

### Skill retention 1 Week after the conclusion of pattern recognition training

Scores of Test 1 (the first test session occurred immediately after training) and Test 2 (the second test session occurred one week later) are shown in Table 3. For Subject TR3, dexterity as measured by the JTHF writing test decreased. Scores of other measures of dexterity (Box and Blocks and other JTHF tests) as well as the AM-ULA were within the margin of error (MDC 90). Scores of the clothespin task decreased for the basic test, but remained the same for dual task performances.

**Table 3:** Change in PR control performance one week after ending PR training

Evaluation		Subject TR3			Subject TR4		
		Test 1	Test 2	Change after 1 week	Test 1	Test 2	Change after 1 week
Box and Blocks		5	3	−2.00	7	15	6
JTHF Tests (items/sec)	Writing	0.44	0.24	−0.20^a^	0.47	0.84	0.38^a^
	Page Turning	0.09	0.07	−0.03	0.21	0.10	−0.11^a^
	Small Objects /sec	0.03	0.03	0.00	0.08	0.05	−0.03
	Simulated Feeding	0.14	0.13	−0.01	0.09	0.07	−0.02
	Stacking Checkers	0.14	0.04	−0.09	0.14	0.17	0.03
	Moving Light Cans	0.11	0.09	−0.02	0.38	0.45	0.07
	Moving Heavy Cans	0.14	0.08	−0.06	0.30	0.32	0.02
Clothespin Relocation (# pins moved)		4	3	1	6	3	−3
Clothespin Relocation with counting backwards		2	5	+ 3	6	5	1
Clothespin Relocation with semantic fluency		5	4	1	4	6	2
AM-ULA		15.0	13.8	−1.2	15.00	13.3	−1.67

^a^Differences between test periods exceeds known MDC90

For Subject TR4, dexterity as measured by the Box and Block increased by 6 blocks (MDC 90 is 6.49). Scores of the JTHF writing test also improved, while the clothespin relocation test showed a decline in performance for the basic task, and one dual task condition (counting backwards), but an improvement in the other dual condition (semantic fluency). Scores of the AM-ULA decreased, but not beyond the margin of error (MDC 90).

### Rate of skill acquisition

**Fig. 3:**
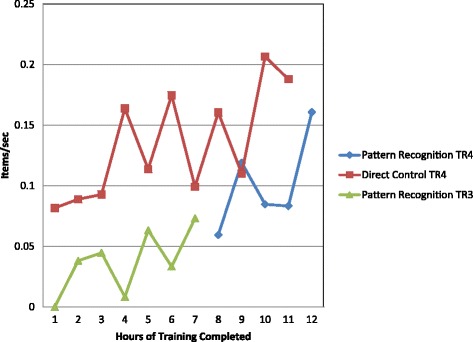
Hourly performance for page-turning task

As shown in Fig. 3, TR4 improved steadily in JTHF page turning with DC over training hours. For PR control, performance improved between the 8th hour of training and the final hour of training. For TR4, DC performance surpassed PR control performance at every testing interval. However, for Subject TR3, who had 7 h of PR control training, there was a pattern of erratic performance, but a trend towards improvement over time. All 7 JTHF page-turning results for TR3 were collected on different days and were collected after 1 h of training. Therefore, neither fatigue nor re-learning are expected to have affected the JTHF page-turning and results in Fig. 3. These findings suggest that for subject TR3, skill performance did not improve significantly with training hours.

## Discussion

This study directly compared self-report and performance outcomes between EMG DC and EMG PR control immediately after training and one week after training ended in a single user who controlled a 2-DOF prosthesis. Additionally, this study examined the change in outcomes 1 week after PR training and the rate of skill acquisition in 2 subjects with TR amputation. Overall, the two control strategies yielded very similar outcomes, with no overwhelming superiority of one method over the other. That said, outcomes for about a quarter of test metrics favored DC control.

Although there was a steady improvement in PR control over training and evidence that control (as measured by a single JTHF page-turning test) had plateaued, the decline in dexterity noted in both subjects one week after training with EMG PR ended suggests that additional training may have been needed to retain newly acquired control gained during training sessions. Given our limited sample size, these results should be considered preliminary. Further evaluation of retention of skill acquisition on more upper limb amputees for EMG PR use is needed to inform the future patient training protocol when EMG PR becomes more widely adopted.

There are several factors that may have contributed to the finding in favor of DC in 26% of test metrics. The subject used DC on a daily basis for only 1-DOF hand control. Although she had to learn how to use DC to switch control mode and how to use hand open/close intent to drive hand rotation, we speculated that she was probably more familiar and comfortable with DC and that might bias the findings towards DC. Second, we found that all subjects initially had difficulties attempting some motion in their missing limb and in creating clearly distinguishable EMG patterns between two motions (e.g. EMG patterns for hand open and supination were often overlapped). This may have been caused by the absence of peripheral structures in the musculoskeletal system, limitations in available muscles in the forearm, inaccessibility of deep residual muscles via surface EMG recordings, and/or neural plasticity that interfered with appropriate recruitment of residual musculature when imagining movement of the phantom limb. After training, both subjects improved EMG PR control, but may have used strategies (such as imagining thumb extension while attempting supination) in order to produce distinguishable EMG patterns. Such an approach required the subjects to learn and remember the strategies applied, and may have led to non-intuitive control of the prosthesis (counter to one supposed benefit for EMG PR control). Solutions such as better neural interfaces (e.g. targeted muscle reinnervation [20] or intramuscular EMG recordings [51]) might be combined with EMG PR to further improve intuitive prosthesis control. Additionally, longer training periods or additional conditioning of residual muscles’ activation should be considered in EMG PR control training to accelerate the user’s learning of EMG PR-based prosthesis control. Although we had a training termination rule that was based on functional performance, it is possible that our subjects would have benefitted from additional EMG PR exposure and training.

To our knowledge, this study is only the second published paper that directly compares EMG PR to DC for transradial prostheses. A recent study, published by Kuiken et al. [30], has been important to the field, but is insufficient evidence to generalize conclusions about the superiority of direct control or EMG PR. Therefore, our study makes a novel contribution to the literature. In contrast to the earlier paper, we found no superiority of EMG PR over DC, and an advantage of DC in 26% of tests. However, our study design and procedures differed substantially from the earlier paper; Kuiken et al. compared control of a 3 DOF prosthesis (hand open/close, wrist pronation/supination and wrist flexion/extension) controlled by EMG PR and EMG DC in the laboratory, and their study included 4 weeks of use in a home setting [30]. They reported no difference in performance metrics after laboratory training, but superiority of PR control after home use in several tasks that required wrist flexion, including the SHAP, Clothespin relocation, and Cubbies task. In contrast, our experimental prosthesis was only 2 DOF (hand open/close and pronation/supination because of their availability in the current market) and, thus, was less complex to control. This result is actually not surprising because the difference between EMG PR and DC starts to manifest when the included prosthesis function increases. When the operation function is limited to hand open/close (1DOF) only, although the algorithm used for EMG PR and DC are different, the prosthesis operation from the user’s point of view is not much different. For both cases, the users need to attempt moving missing hand open or close in order to operate the prosthetic hand. The more the effort exerted by the user (related to higher magnitude of EMG signals), the faster the prosthetic joint moves. The users need to relax all the muscles in order to stop prosthesis hand motion. Therefore, one can simply treat EMG thresholding methods used in DC as a simple version of EMG pattern classification when operating 1DOF hand. The user’s operation of hand open/close via EMG signals is natural for both algorithms. The benefit of EMG PR control in a 2 or more DOF device is that the user does not need to switch the control joint and instead directly attempts the intended motion. In addition, the user’s attempted joint motion is directly mapped to the motion of prosthetic joints, which is different from DC control that non-intuitively maps hand open/close intent to other joint motions (e.g. hand rotation). Given the conflicting results between studies, we hypothesize that EMG PR control may be more beneficial than DC when the myoelectric prosthesis includes 3 or more powered joints. In contrast, for 2 DOF prostheses, EMG PR control might not add additional clinical value compared to DC. A recent study by Hargrove et al. compared EMG PR and direct control of a 3 DOF prosthesis after 6–8 weeks of home use for transhumeral amputees who had targeted muscle reinnervation [52]. That study reported improved performance on two tests of dexterity and hand function. Another possibility is that our subject did not optimize their performance with the PR system. Kuiken et al. [30] included home use experience in their protocol. It is possible that we may have seen similar differences in outcomes given greater practice achieved through home use experience.

Clearly, further studies that include larger samples are needed to compare 2 DOF prosthesis control strategies after in-laboratory training and with home experience. That said, we recognize that large-scale studies with transradial amputees are time-intensive and challenging due to the small size and geographic dispersion of the amputee population. A multi-site study would yield a larger sample size, but would incur considerable cost and require a substantial external funding. Comparisons of results of case studies from small-scale research endeavors, particularly if they use standardized experimental protocols to evaluate the performance of PR control and DC, may be a viable means of summarizing data across multiple studies. This research structure would lower the cost of research, allowing it to be shared among multiple funding sources. By analyzing the impact of recent advancements in prosthetics research on clinical viability and at-home success of amputees, technological developments, which more meaningfully impact amputees, may be realized.

Based on the experiences, results, and lessons learned in this study, we have several suggestions for future trials that aim to compare these two myoelectric control methods for transradial prostheses. First, we believe that it is important to consider the degrees of freedom in the prosthesis that need to be controlled, and that studies be conducted in using both 2 and 3 DOF devices. Second, as aforementioned, training protocols for amputees using EMG PR control may need to include additional sessions or training methods for conditioning EMG activation patterns because the amputees’ ability to generate consistent, distinct EMG activation patterns among intended motions is crucial for successful EMG PR control. Third, engineering efforts are needed to collect data related to the control system performance (such as calibration frequency and duration, pattern recognition errors, reliability, etc.) in order to understand the challenges in the engineering machines that contribute to user performance. Finally, we recommend that a standardized experimental protocol be adopted for comparisons between PR control and DC so that results from different research centers may be combined, yielding a larger effective sample size.

Our study is limited by the small sample size and descriptive analyses. Nevertheless, our results provide important evidence and illustrate a research, evaluation and training protocol, which could be reproduced in future case studies or larger-scale studies. Given the challenges in recruiting and training this patient population and the paucity of literature directly comparing control strategies, we believe that this study makes an important contribution to the literature.

## Conclusions

This case study compared EMG PR control with conventional direct myoelectric control, in a cross-over study involving a single subject with TR amputation, and compared skill retention and rate of skill acquisition in 2 subjects operating a 2 DOF transradial prosthesis. In the cross-over study, we observed that the two control strategies produced nearly identical outcomes for 75% of the metrics administered. There were differences favoring DC over PR control in 2 measures of dexterity, one measure of dexterity with cognitive load, and one measure of self-reported function. Both subjects showed decline in dexterity one week after PR training ended. Findings related to rate of skill acquisition varied considerably by subject, with one subject showing improvement after each testing interval and the other showing erratic performance.

Our findings differed from those reported in a previous home trial which included three transradial amputees using a 3 DOF prosthesis and suggested more favorable outcomes of EMG PR control, particularly for tasks involving wrist flexion. This difference suggests that additional research comparing these two myoelectric control methods in both 2 and 3 DOF devices is needed to understand the relative benefits of EMG PR control. The results of our early stage case study provides a preliminary understanding of the comparative benefits of EMG PR control and provides an example of a research design that could be employed in future studies comparing PR and existing clinical prosthesis control.

## Additional file

Additional file 1: Training Protocol. (DOCX 29 kb)

## Abbreviations

AM-ULA: Activities Measure for Upper Limb Amputees
DC: Direct control
DOF: Degree-of-freedom
EMG: Electromyogram
IRB: Institutional review board
JTHF: Jebsen Taylor hand function
LDA: Linear discriminant analysis
MAV: Mean absolute value
MDC: Minimally detectable change
OPUS: Orthotics and prosthetics users survey
PR: Pattern recognition
PSFS: Patient specific functional scale
TAPES: Trinity amputations and prosthetics experience scale
TD: Time-domain
TR: Transradial
UEFS: Upper extremity functional scale
VR: Virtual reality

## Footnotes

1 This procedure is usually called “training procedure for establishing EMG PR-based control”. In order to avoid confusion with the training procedure for healthy human subjects, we refer to this procedure as the “calibration procedure”.
