# Supplementary material for: Evaluation of EMG pattern recognition for upper limb prosthesis control: a case study in comparison with direct myoelectric control
Source: J Neuroeng Rehabil. 2018 Mar 15;15:23. doi: 10.1186/s12984-018-0361-3 (PMC5856206; doi:10.1186/s12984-018-0361-3)
Supplement: Supplementary file 1 — Training Protocol. (DOCX 29 kb) [file 12984_2018_361_MOESM1_ESM.docx]

**Additional file 1: Training Protocol**

**Pattern Recognition Early Training Protocol**

Subjects will be trained in the use of either conventional direct EMG-controlled prosthesis (using 2 EMG sites) or EMG-PR-controlled prosthesis for 10 hours, or more is needed, over a period of approximately 5 training sessions. The training will end when the skill level has plateaued. Every attempt will be made to schedule training visits 3-5x/week. However, the schedule of training visits may vary due to subject and staff availability. Subjects will be given 5 minutes of rest every 30 minutes, or more if necessary**.**

**INITIAL TRAINING VISIT/S**

1. Demonstration and Explanation
   1. Explain/review how pattern recognition works.
   2. If the subject is a current (or former) myoelectric user, help him/her identify and discuss differences between their control set-up and pattern recognition.
2. Controls Training with Virtual Reality Training
   1. Begin with the subject wearing the experimental socket and/or prosthesis attached, but with the prosthesis disabled.
   2. Subject imagines their phantom limb performing each movement and watches results on the VRE.
   3. Ask subject to choose related/intuitive movements that will be easy to remember.
   4. Ask the subject to use their sound hand to show each movement that is being imagined with the phantom limb.
   5. Teaching points:
      - Obtain distinguishable contractions- unique from each other
      - Emphasize the need to pay attention to how the muscle contractions feel to get the consistency of movements/muscle patterns.
      - Avoid increasing muscular force.
      - Avoid making drastic modifications to movements to achieve results.
   6. Practice each movement and view results on screen
      Start simple:

HAND OPEN: Hand at rest, open hand/extend fingers

HAND CLOSE: Hand at rest, make a fist

PRONATION: Start with forearm in neutral, turn palm down

SUPINATION: Start with forearm in neutral, turn palm up

- - - Prompt the subject to perform the full range of each movement.
    - Practice movements first with mirroring with the sound extremity.
    - Repeat movements without mirroring with the sound extremity.
  1. If the subject is having difficulties with consistency or distinguishability of patterns:
     - Isolate wrist and hand movements
     - Experiment with nonessential components of the movement to get clearer signals
     - Rest at least 5 minutes every 30 minutes, more often if subject is fatigued
  2. Once the subject has good distinguishability of movements ask them to describe, in simple words, the movement that they are imagining their phantom limb doing.
     - Record these “cue words” for use in coaching and training.
  3. Practice combinations of movements
- Vary the sequence of the four basic movements.
  1. Practice movements in varying workspaces.
     - Close to the body
     - Extended in front of the body
     - Extended with arms to the side
     - Arms overhead
  2. During practice provide frequent reminders about the consistency of contractions, avoiding “trying too hard”, and request rest if tired. Have subject use the “cue words” they identified in step “g” above.
  3. More practice!

Virtual reality training should continue until the subject is able to demonstrate moderately consistent command of controls for each of the four movements (hand open, hand closed, supination, and pronation).

**TRAINING WITH THE PROSTHESIS ACTIVATED**

Subsequent training will progress from controls review and operation to simple grasp and release activities, to performance of subject-requested activities, to more complex utilization of the prosthesis in unilateral and bilateral tasks. The therapist will use clinical judgment as to the appropriate amount of repetition needed to move from one activity to the next.

1. Check the Controls:

The first training session of each new day begins with a check of control operation. If the subject is having difficulty with the operation of any movement, recalibration by the engineering team or re-donning the prosthetic socket may be necessary.

1. Controls Training:
   1. Controls training with the prosthesis activated involves the same steps described above in the Initial Training Protocol sections e-k.

3. Progress activities following the “generic” training protocol, which is identical for pattern recognition and direct control and described below.

**Pattern Recognition and Direct Controls Training Protocol**

1. Grasp and release training
   1. Grasp and release activities should be initiated in the midline with objects on a tabletop directly in front of the subject. The therapist should utilize a variety of shapes, sizes, and textures of objects that require the subject to select and use each of the grips.

During grasp and release training the therapist should cue subject about the following aspects of Arm control:

- The approach used (pre-positioning of forearm and wrist and shoulder if needed).
- Control of the force of grasp and release.

Grasp and Release should be practiced in a variety of positions

- Close attention must be paid to minimize any awkward or compensatory body motions when subjects approach an object.
  1. Stacking plastic cups:
- Subjects stack large drinking cups in a pyramid, four or five levels high, to reinforce grasp and release and tension control.
  1. Shadow boxing with grasp and release of a pen:
- Progress shadow boxing to having the subject follow the pen, grasp it, release it and give to the therapist.
- Repeat in different planes of motion.

1. Unilateral ADL Task Training: To minimize frustration and enhance learning, task-based activities should not take place until the subject is completely familiar with the control sites and can control the Arm/hand movement in a “natural” way. Ideally, simple task-based activities will begin by the 3rd training visit to minimize boredom, but this may vary by subject.
   1. Everyday task training should include but are not limited to activities from the AM-ULA (except those involving water).
   2. While in a natural environment, the “sound hand”, in a unilateral amputee, will probably be used to accomplish many of these activities; however, for the purposes of this study, the subject should be encouraged to perform these activities using their prosthetic side.
   3. During these training activities, the therapist should pay careful attention to the subject’s posture and the positioning of the prosthesis. The therapist should coach subjects to avoid compensatory movements and to use the full capabilities of the prosthesis.
2. Bilateral ADL Task Training: Bilateral activities reinforce the use of the prosthesis as a “functional assist.”
   1. The therapist needs to pay attention to body posture and Arm/hand positioning and coach the subject to avoid compensatory movements.
3. Advanced Training: Advanced training should only begin when the therapist is comfortable with the subject’s level of performance in less complex activities.
   1. Advanced training should include the performance of short-term projects, vocational, avocational and recreational tasks, such as games, including Jenga, Connect Four, Grooved Pegboard and Operation.
   2. The selection of activities should be based on the specific interests of the subject and become a “fun” and goal-oriented stage of training. Subjects have identified a set of activities in the Patient Specific Functional Scale (PSFS) at the beginning of the study. We recommend that you include training in these activities, if possible.
4. These activities might include, but are not limited to:

Preparing a simple meal of subject’s choosing.
Completing a small woodworking or model building project.
Putting a golf ball.
Playing an instrument.

1. The therapist and subject should try to identify an advanced activity at least one visit ahead of time so that all necessary supplies and equipment can be gathered.
2. Training should also include some time for the subject to use the prosthesis without any instruction. This time will help the subject experiment with arm motions and simple activities of his/her own choosing. During this time, the subject should be encouraged to try the activity independently and without any coaching or teaching, except as necessary to maintain safety.
